# Widespread Sexual Dimorphism in the Transcriptome of Human Airway Epithelium in Response to Smoking

**DOI:** 10.1038/s41598-019-54051-y

**Published:** 2019-11-26

**Authors:** Chen Xi Yang, Henry Shi, Irving Ding, Stephen Milne, Ana I. Hernandez Cordero, Cheng Wei Tony Yang, Edward Kyoo-Hoon Kim, Tillie-Louise Hackett, Janice Leung, Don D. Sin, Ma’en Obeidat

**Affiliations:** The University of British Columbia Centre for Heart Lung Innovation, St. Paul’s Hospital, Vancouver, BC Canada

**Keywords:** Data mining, Microarrays, Chronic obstructive pulmonary disease, Molecular medicine

## Abstract

Epidemiological studies have shown that female smokers are at higher risk of chronic obstructive pulmonary disease (COPD). Female patients have worse symptoms and health status and increased risk of exacerbations. We determined the differences in the transcriptome of the airway epithelium between males and females, as well the sex-by-smoking interaction. We processed public gene expression data of human airway epithelium into a discovery cohort of 211 subjects (never smokers n = 68; current smokers n = 143) and two replication cohorts of 104 subjects (21 never, 52 current, and 31 former smokers) and 238 subjects (99 current and 139 former smokers. We analyzed gene differential expression with smoking status, sex, and smoking-by-sex interaction and used network approaches for modules’ level analyses. We identified and replicated two differentially expressed modules between the sexes in response to smoking with genes located throughout the autosomes and not restricted to sex chromosomes. The two modules were enriched in autophagy (up-regulated in female smokers) and response to virus and type 1 interferon signaling pathways which were down-regulated in female smokers compared to males. The results offer insights into the molecular mechanisms of the sexually dimorphic effect of smoking, potentially enabling a precision medicine approach to smoking related lung diseases.

## Introduction

Chronic obstructive pulmonary disease (COPD) affects more than 300 million people and is the 3^rd^ leading cause of death worldwide^1^. In Canada, COPD is the leading cause of hospitalization, accounting for 80,000 hospital admissions per year^2^. While historically men used to smoke more, there has been a marked rise in the prevalence of smoking among women since the 1960’s, leading to a sharp increase in the burden of COPD among women. In the year 2000 the number of women dying of COPD surpassed that of men in the United States^3^, and in Canada recent estimates suggest that there are approximately 85% more female COPD patients than male patients^4^. Our group has reported previously that female smokers experience a heightened risk of COPD, particularly following menopause^5^. These data have been validated in a study involving 149,075 women and 100,252 men in the UK Biobank^6^. For the same severity of airflow obstruction, female patients have increased shortness of breath, poorer quality of life and health status, and greater functional impairment than male patients^7^. Furthermore, women with COPD are more likely to experience increased risk of exacerbations^7^.

There is a growing body of evidence from histological, imaging and animal models to support the notion that females have more airways disease and less emphysema compared to male COPD patients^8,9^. In a murine model where male and female (ovariectomized and ovary-intact) mice were exposed to smoke for 6 months, the female mice with intact ovaries had significantly thicker small airway walls but had similar degrees of emphysema compared with male or ovariectomized female mice^10^.

Females are also thought to have augmented xenobiotic metabolism of inhaled chemicals such as nicotine^11^. Since some metabolites of nicotine may be toxic, the sex-related differences in xenometabolism maybe an important driver of increased female susceptibility to COPD^12,13^. In addition, we have shown *in vitro* that females have enhanced ability to produce oxidants in the airways in response to cigarette smoking, leading to increased airway damage^14,15^.

To further understand the mechanisms underlying the sexual dimorphism in the pathogenesis of smoking related lung diseases, we examined the effects of sex, and its interaction with cigarette smoking, on the transcriptome of human airway epithelial cells.

## Results

**Figure 1:**
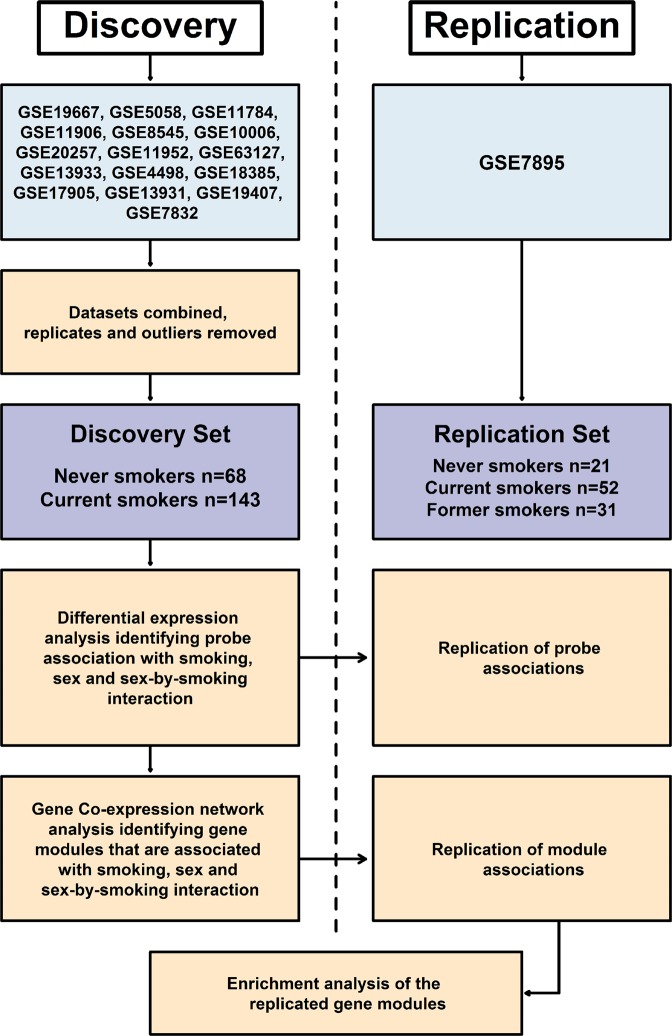
Overall study design.

The overall study design is shown in Fig. 1. The demographics of the discovery and replication cohorts are described in Table 1. The discovery set included 68 never smokers and 143 current smokers while the replication set included 21 never smokers, 31 former smokers and 52 current smokers. There were no statistically significant differences in age or pack-years between female never smokers, female current smokers, male never smokers and male current smokers in the discovery or the replication sets (all Bonferroni adjusted p-values > 0.05). We also used a third dataset (GSE37147) for potential replication although it only contained data on former and current smokers (i.e. there were no never-smokers). However, compared to the discovery and replication sets, all except male former smokers in GSE37147 were significantly older (all Bonferroni adjusted p-values < 0.05). The male current smokers in GSE37147 also had increased pack-years compared to those in the discovery or the replication sets (Bonferroni adjusted p-values < 0.05). The female current smokers in GSE37147 had increased pack-years compared to those in the discovery set (Bonferroni adjusted p-value = 9.04 × 10^−03^) but not to those in the replication set (Bonferroni adjusted p-value = 0.95).

**Table 1:** Demographics of subjects in the discovery and the replication set.

Sex	Discovery Set				Replication Set						GSE37147			
	Female n = 70, 33.2%		Male n = 141, 66.8%		Female n = 25, 24.0%			Male n = 79, 76.0%			Female n = 103, 43.3%		Male n = 135, 56.7%	
Smoking Status	NS n = 26, 37.1%	CS n = 44, 62.9%	NS n = 42, 29.8%	CS n = 99, 70.2%	NS n = 5, 20.0%	FS n = 10, 40.0%	CS n = 10, 40.0%	NS n = 16, 20.3%	FS n = 21, 26.6%	CS n = 42, 53.1%	FS n = 54, 52.4%	CS n = 49, 47.6%	FS n = 85, 63.0%	CS n = 50, 37.0%
Age	(38.0, 23.5)	(46.0, 12.5)	(39.5, 11.5)	(45.0, 7.5)	(28.0, 7.0)	(44.0, 11.8)	(40.5, 13.5)	(29.5, 14.5)	(63.0, 18.0)	(47.5, 25.3)	(66.0, 6.98)	(63.7, 6.5)	(66.3, 8.7)	(61.1, 7.37)
Pack years	(0.0, 0.0)	(28.5, 16.8)	(0.0, 0.0)	(26.0, 21.8)	(0.0, 0.0)	(6.0, 25.8)	(24.0, 17.1)	(0.0, 0.0)	(35.0, 52.0)	(25.0, 39.4)	(42.0, 15.0)	(47.1, 15.1)	(47.6, 19.9)	(43.2, 13.8)
COPD	n = 0	n = 11	n = 0	n = 31	NA	NA	NA	NA	NA	NA	n = 21	n = 14	n = 36	n = 16

Descriptive statistics are shown as (median, interquartile range). NS = Never Smoker, FS = Former Smoker, CS = Current Smoker.

### Prediction of sex in the replication set

The best-performing elastic net-regularized logistic regression model consisted of three probes and together they demonstrated 100% cross-validated area under the receiver-operator characteristics curve (AUC) in the training dataset. These three probes corresponded to *RPS4Y1*, *DDX3Y* and *KDM5D* genes, which are located on the Y-chromosome. The model was then evaluated in the testing dataset and also showed perfect ability to discriminate with an AUC of 100% (Supplementary Fig. 2A,B). After confirming the model performance, we applied it to the replication set to classify the subjects into 79 males and 25 females (Supplementary Fig. 2C).

### Differential expression with sex and smoking

As shown in the principal component analysis (PCA) plot in Supplementary Fig. 1E, there was clear clustering in the gene expression profiles of the discovery set, the replication set, and GSE37147. The differences may be related to different microarray platforms that were used and different generations of the airway that were sampled across the three studies.

**Figure 2:**
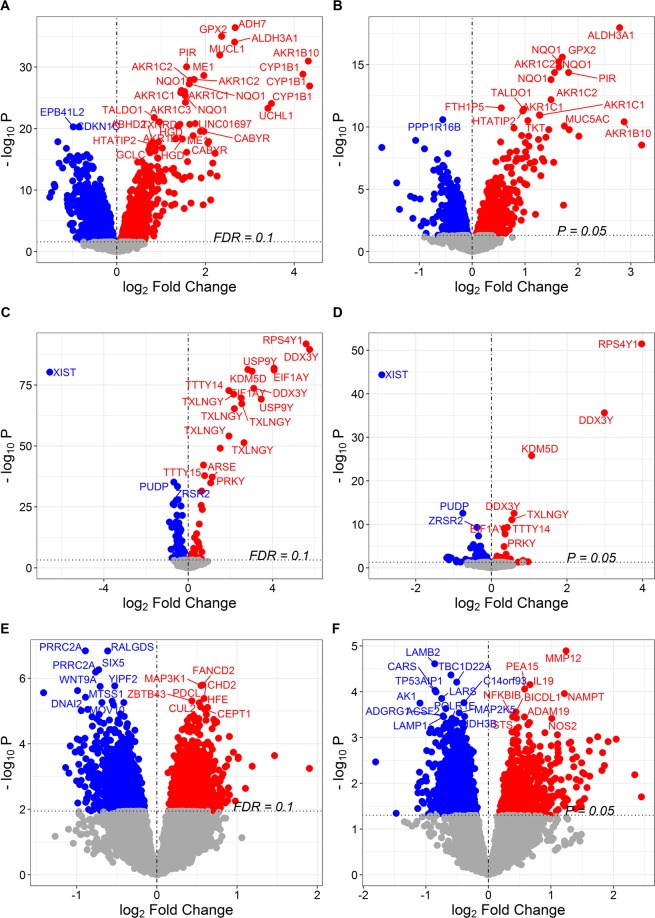
Volcano plots showing probe-phenotype associations. (**A**) Probe associations for smoking status in the discovery set. (**B**) Probe associations for smoking status in the replication set. (**C**) Probe associations for sex in the discovery set. (**D**) Probe associations for sex in the replication set. (**E**) Probe associations for sex-by-smoking interaction in the replication set. (**F**) Probe associations for sex-by-smoking interaction in the discovery set. Probes shown in red were up-regulated in current smokers and probes shown in blue were down-regulated in current smokers in (**A**) and (**B**). Probes shown in red were up-regulated in males and probes shown in blue were down-regulated in males in (**C**) and (**D**). Probes shown in red exhibit a positive sex-by-smoking interaction in male current smokers while the probes shown in blue exhibit a negative sex-by-smoking interaction in male current smokers in (**E**) and (**F**).

In the discovery set, 8,000 probes, corresponding to 5,466 genes, were differentially expressed between never and current smokers after adjustment for sex, age, COPD, ethnicity and pack-years of smoking (FDR < 0.1) and 735 of these genes were replicated in the replication set (at a threshold of P < 0.05) (Fig. 2A,B). To identify and replicate genes, an FDR < 0.1 was used in the hypothesis-free discovery analyses. For replication, a gene was considered “replicated” if any of the probes showed differential expression between never and current smokers in the replication cohort at P < 0.05 and with the same direction of effect. The top three replicated genes associated with smoking were *ADH7* (FDR = 1.15 × 10^−32^), *GPX2* (FDR = 1.43 × 10^−31^) and *ALDH3A1* (FDR = 8.56 × 10^−31^), which were all up-regulated in current smokers. We also validated the discovery in a second replication set GSE37147; out of the 735 replicated genes, 526 were further replicated in GSE37147 study of former versus current smokers.

**Figure 3:**
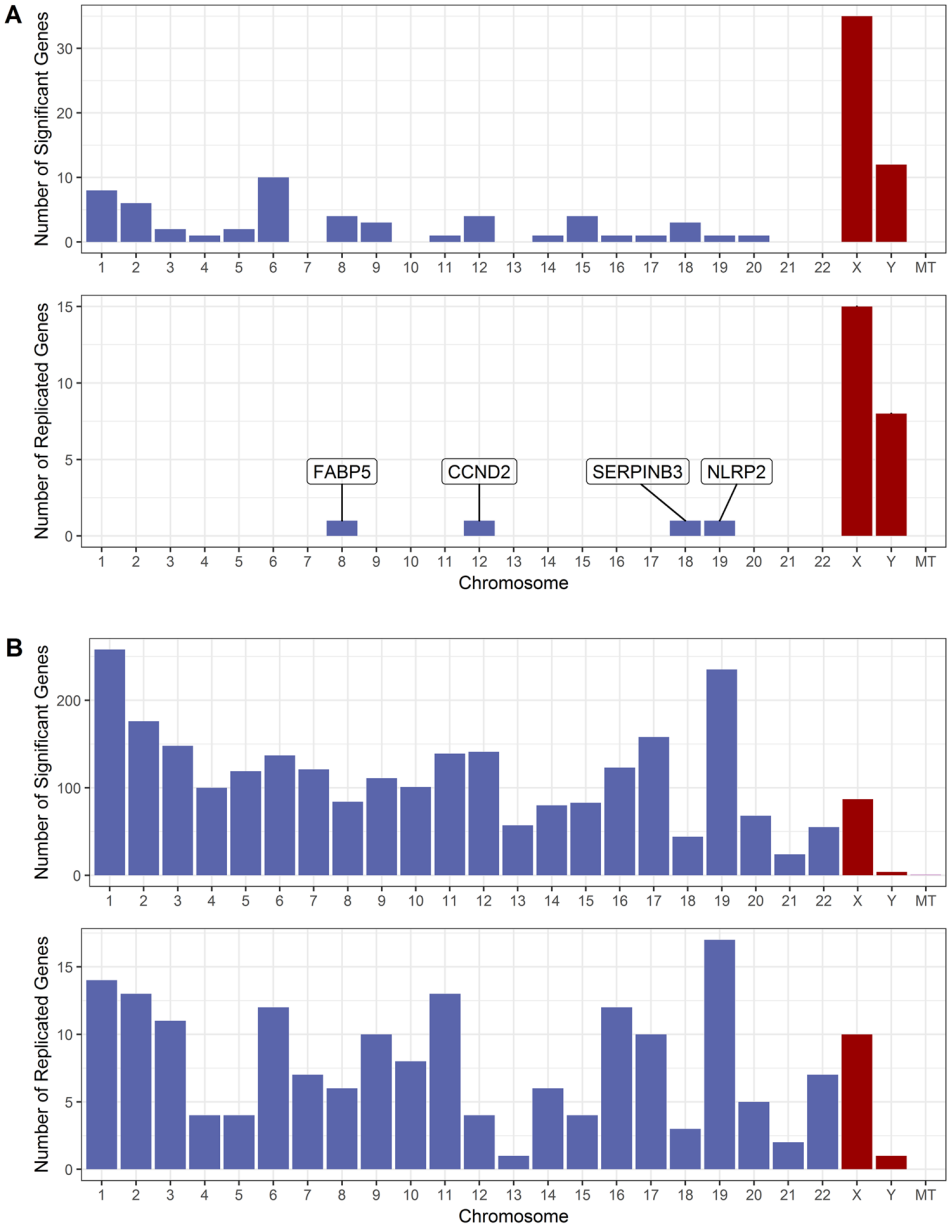
Chromosome distribution of genes that were associated with sex or sex-by-smoking interaction. (**A**) Top: Chromosome distribution of genes associated with sex in the discovery set (FDR < 0.1). Bottom: Chromosome distribution of the replicated genes for sex. (**B**) Top: Chromosome distribution of genes associated with sex-by-smoking interaction in the discovery set (FDR < 0.1). Bottom: Chromosome distribution of the replicated genes for sex-by-smoking interaction. Abbreviation used in the figure: MT = mitochondrion.

A total of 169 probes, corresponding to 100 genes, were differentially expressed between males and females after adjustment for smoking status, age, ethnicity and pack-years (FDR < 0.1), and 27 of these genes were replicated in the replication set (P < 0.05) (Fig. 2C,D). Four of the replicated genes, *CCND2*, *FABP5*, *NLRP2*, and *SERPINB3*, are on the autosome (Fig. 3A). *CCND2* and *NLRP2* were down-regulated in males while *FABP5* and *SERPINB3* were up-regulated in males. The top three genes differentially expressed between males and females were *RPS4Y1* (FDR = 3.98 × 10^−88^), *DDX3Y* (FDR = 4.33 × 10^−86^) and *EIF1AY* (FDR = 6.79 × 10^−82^), which are located on the Y-chromosome (all three genes were up-regulated in males). Additional evaluation using GSE37147 replicated 22 out of the 27 replicated genes, including *CCND2* and *NLRP2*.

The complete lists of genes that were significantly associated with smoking status and sex are shown in Supplementary Tables 2 and 3, respectively.

### Sexually dimorphic gene expression changes associated with smoking

**Figure 4:**
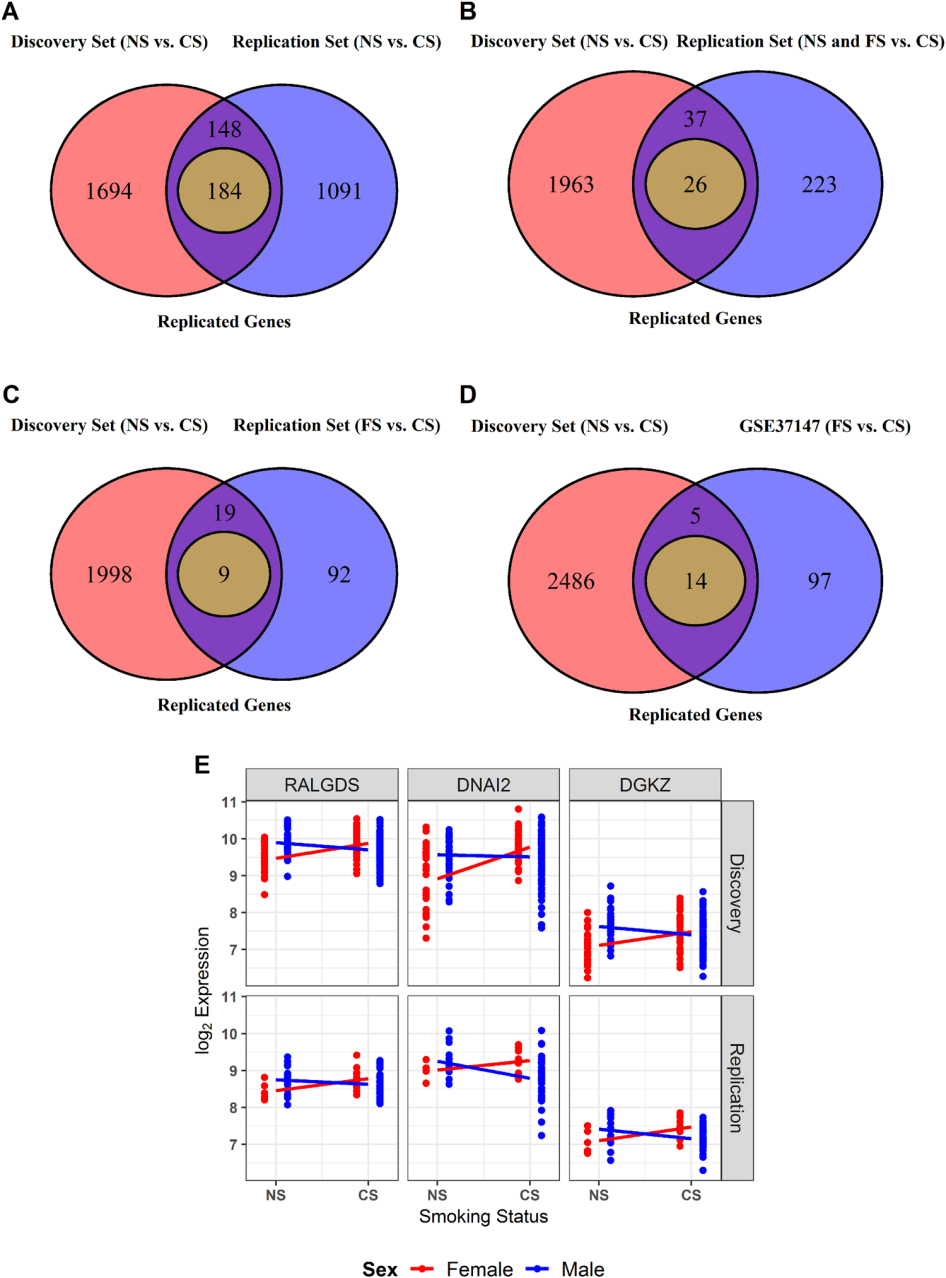
Replication of genes that were associated with sex-by-smoking interaction. (**A**) Venn diagram showing the number of associated genes in the discovery set (NS vs. CS) and in the replication set (NS vs. CS). (**B**) Venn diagram showing the number of associated genes in the discovery set (NS vs. CS) and in the replication set (NS and FS combined together vs. CS). (**C**) Venn diagram showing the number of associated genes in the discovery set (NS vs. CS) and in the replication set (FS vs. CS). (**D**) Venn diagram showing the number of associated genes in the discovery set (NS vs. CS) and in GSE37147 (FS vs. CS). (**E**) Gene expression of the top 3 replicated genes for sex-by-smoking interaction in the discovery and the replication set. Abbreviations used in the figure: NS = Never Smoker, FS = Former Smoker, CS = Current Smoker.

Having identified genes differentially expressed according to sex and smoking status, we next identified sexually dimorphic genes in response to smoking. In the discovery set, 3,416 probes corresponding to 2,654 genes were associated with sex-by-smoking interaction (FDR < 0.1) (Fig. 2E). In the replication set, 1,737 probes corresponding to 1,423 genes were associated with sex-by-smoking interaction (P < 0.05) (Fig. 2F). A total of 184 genes showed differential expression between never and current smokers in both datasets and within the same direction (Fig. 4A). The top three replicated genes were *RALGDS* (FDR = 2.19 × 10^−03^), *DNAI2* (FDR = 9.84 × 10^−03^) and *DGKZ* (FDR = 1.48 × 10^−03^). The sexually dimorphic genes in response to smoking were not restricted to the XY chromosome; 96.5% of the associated genes in the discovery set and 94.0% of the 184 replicated genes were located on the autosome (Fig. 3B). The lists of probes that were associated with the sex-by-smoking interaction term are shown in Supplementary Table 4 in the discovery and the replication sets. Interestingly, the number of replicated genes decreased to 26 when we combined the never and former smokers together and compared them with the current smokers in the replication set, despite the increase in sample size (Fig. 4B). The number of replicated genes decreased to 9 if we compared the former smokers to the current smokers in the replication set (Fig. 4C). Similarly, when GSE37147 (containing current and former smokers but no never-smokers) was used as the replication set, only 14 genes were replicated (Fig. 4D). Figure 4E shows the expression of the top three replicated genes in both the discovery and the replication set (never vs. current smokers). If we use GSE37147 to further replicate the 184 replicated genes, only one gene, *PLCB2*, was also replicated in GSE37147.

### Module-level associations

Based on the expression of 12,537 genes in the discovery set, 37 gene modules were constructed. The module sizes ranged from 17 genes in module 36 to 3,822 genes in module 01. Eleven modules were significantly associated with smoking status (never vs. current smokers) after adjusting for age, sex, ethnicity and pack-years of smoking (FDR < 0.1) in the discovery set. Four of these modules – module 22, module 11, module 17, and module 15 – were successfully replicated in the replication set (never and former vs. current smokers) at a threshold of P < 0.05.

Only module 36 was associated with sex (FDR < 0.1) in the discovery set and it was also replicated in the replication set at a threshold of P < 0.05.

**Figure 5:**
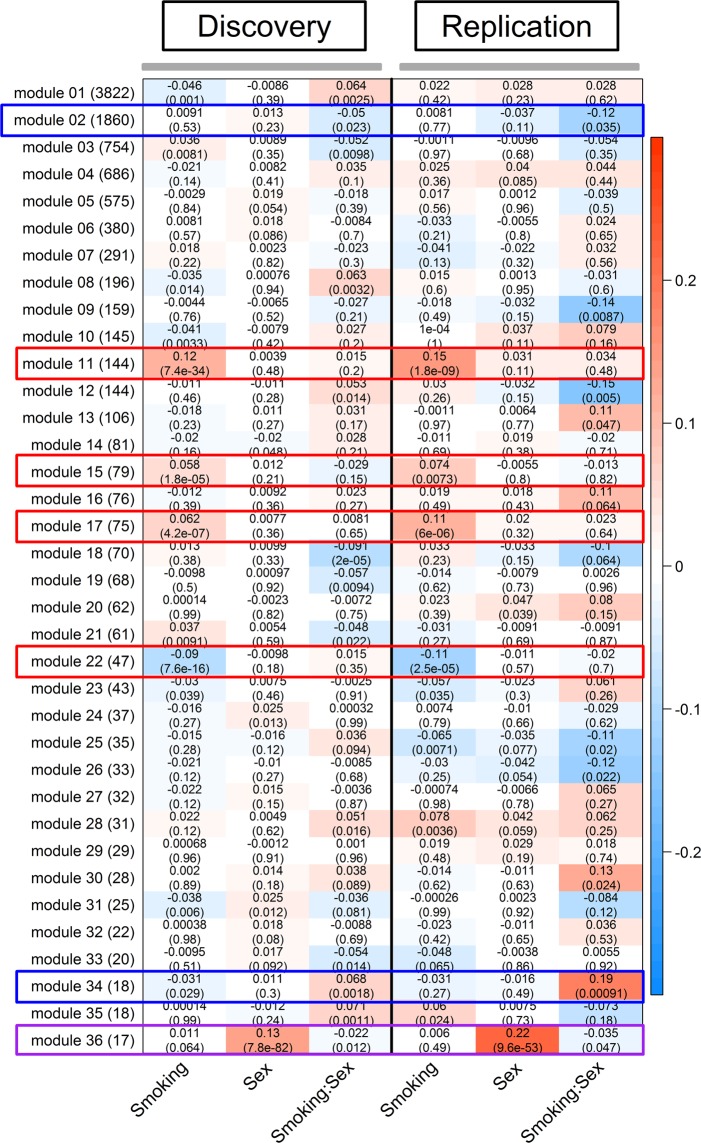
Module-level associations in the discovery and replication sets. The rows represent the gene modules and the sizes of the modules are shown within parentheses next to the module names. The columns represent the phenotypes. In each cell, the number at the top is the linear regression coefficient and the number in parentheses is the corresponding p-value. The color of the cell is proportional to the regression coefficient. From column 1 to column 3 of both the discovery and the replication sets, the red color indicates (1) up-regulation in current smokers, (2) up-regulation in male subjects, and (3) a positive sex-by-smoking interaction effect in male current smokers. The replicated modules have been highlighted (red for smoking effect, blue for smoking-by-sex interaction and purple for both sex effect and smoking-by-sex interaction).

Thirteen modules were associated with sex-by-smoking interaction in the discovery set (FDR < 0.1). Three modules – module 02, module 34 and module 36 – were successfully replicated in the replication set (P < 0.05). Module 34 and module 36 were relatively small in size with 18 and 17 genes, while module 02 included 1,860 genes. The module-phenotype association along with the module sizes for the replicated modules are shown in Fig. 5. The associations between the modules and the phenotypes are shown in Supplementary Table 5.

### Gene set variation analysis (GSVA)

In addition to the gene-level and the WGCNA network-level differential expression analyses, we also performed GSVA to replicate the identified gene signatures in the replication cohort. As mentioned previously we have identified 2,654 genes and 13 gene modules associated with sex-by-smoking interaction using gene- and network-level analysis, respectively. The 2,654 genes were considered as one gene set, and each of the 13 modules were considered as a separate gene set. Using GSVA, we found that the gene set with the 2654 genes was not significantly related to sex-by-smoking interaction in the replication cohort (P = 0.660) while module 34 and module 28 were significantly associated with sex-by-smoking interaction (P = 0.016 and P = 0.039, respectively) in the replication cohort.

### Enrichment in biological processes

An FDR cutoff of 0.1 was used to identify Gene Ontology (GO) biological processes that were associated with the replicated modules. Among modules that were associated with smoking status, module 22 genes were associated with protein complex assembly and the top genes with the highest module membership (MM) in this module were *NPAS3, CYP4X1* and *PGRMC1*. Module 11 genes were associated with metabolic processes, responses to oxidative stress and homeostasis and the top members of this module were *AKR1B10*, *GPX2*, *AKR1C2*. Module 15 genes were related to cornification, protein glycosylation and viral entry into host cell. The top members of the module 15 module are *ST14*, *HM13*, *TMPRSS4*. No significant pathways were enriched in module 17 and the top members of this module were *CEACAM5*, *GALE* and *AGR2*.

Of the three modules associated with sex-by-smoking interaction, all the genes in module 36 belonged to the X and Y chromosomes and showed enrichment in translational initiation, protein targeting to endoplasmic reticulum, and histone demethylation. The top members of module 36 were *DDX3Y*, *KDM5D* and *USP9Y*. This module was also associated with sex. For the remaining two modules, module 34 showed strong association with defense responses to virus, regulation of innate immune response, membrane fusion, and Th2 cell cytokine production. The genes with the highest MM in module 34 were *DDX60*, *SAMD9* and *IFI44*. Module 02 was related to autophagy, biological processes of mitochondrion, cellular response to oxidative stress, and negative regulation of the MAPK signaling cascade. The top members of module 02 were *RALY*, *UQCRC1* and *MAP2K2*. Table 2 shows the top GO biological processes enriched in the replicated modules and Supplementary Table 6 shows the complete enrichment results for the replicated modules. Supplementary Table 7 shows the list of top 10 genes with the highest membership in each module.

**Table 2:** Top GO biological processes enriched in each replicated module. The associated effect is shown in brackets.

ID	GO biological process	FDR
**module 22 (smoking)**		
GO:0031333	negative regulation of protein complex assembly	1.77 × 10^−02^
**module 11 (smoking)**		
GO:1901661	quinone metabolic process	6.30 × 10^−06^
GO:0006081	cellular aldehyde metabolic process	6.30 × 10^−06^
GO:0019748	secondary metabolic process	1.86 × 10^−05^
GO:0098869	cellular oxidant detoxification	2.05 × 10^−05^
GO:0034754	cellular hormone metabolic process	4.09 × 10^−05^
**module 17 (smoking)**		
No significant enrichment.		
**module 15 (smoking)**		
GO:0070268	cornification	4.60 × 10^−02^
GO:0006486	protein glycosylation	4.60 × 10^−02^
GO:0043413	macromolecule glycosylation	4.60 × 10^−02^
GO:0016266	O-glycan processing	4.60 × 10^−02^
GO:0046718	viral entry into host cell	4.60 × 10^−02^
**module 36 (sex and smoking:sex)**		
GO:0006413	translational initiation	1.12 × 10^−03^
GO:0006687	glycosphingolipid metabolic process	4.80 × 10^−02^
GO:0006614	SRP-dependent cotranslational protein targeting to membrane	4.80 × 10^−02^
GO:0006613	cotranslational protein targeting to membrane	4.80 × 10^−02^
GO:0045047	protein targeting to ER	4.80 × 10^−02^
**module 02 (smoking:sex)**		
GO:0006914	autophagy	2.91 × 10^−06^
GO:0061919	process utilizing autophagic mechanism	2.91 × 10^−06^
GO:0000422	autophagy of mitochondrion	9.42 × 10^−05^
GO:0061726	mitochondrion disassembly	9.42 × 10^−05^
GO:0035966	response to topologically incorrect protein	4.82 × 10^−04^
**module 34 (smoking:sex)**		
GO:0009615	response to virus	1.29 × 10^−14^
GO:0045071	negative regulation of viral genome replication	2.53 × 10^−07^
GO:0043900	regulation of multi-organism process	3.25 × 10^−07^
GO:0019079	viral genome replication	6.30 × 10^−06^
GO:0060337	type I interferon signaling pathway	2.52 × 10^−03^

In addition to the modules that were replicated through WGCNA, module 28, which was associated with sex-by-smoking interaction and replicated using GSVA, was associated with the amino sugar biosynthetic process (FDR = 0.080).

**Figure 6:**
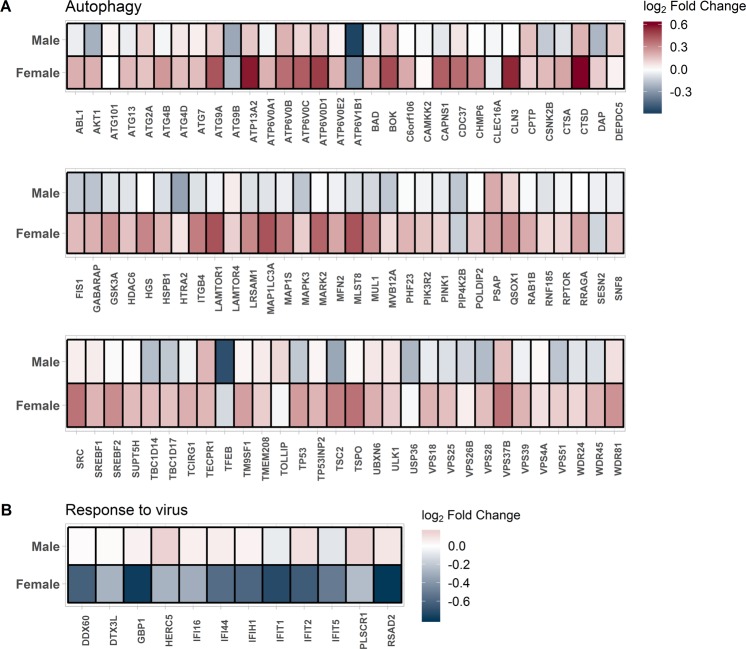
Sexually dimorphic effect of smoking on gene expression of genes in the top GO biological process in (**A**) module 02 and (**B**) in module 34. The red color indicates up-regulation in current smokers and the blue color indicates down-regulation in current smokers.

In Fig. 6, the gene expression changes for genes in the significant pathways (autophagy and response to virus) are shown in males and females separately in response to smoking. While genes in autophagy pathway show little change in males, they were significantly up-regulated in females in response to smoking. On the other hand, genes in the viral response pathway showed little change in males but were significantly down-regulated in females in response to smoking.

### Enrichment in transcription factor binding site (TFBS)

A FDR cutoff of 0.05 and an odds ratio > 2 were used to identify TFBSs that were enriched in genes in the replicated modules. For modules that were associated with smoking status, 13 TFBSs were enriched in module 22, 8 TFBSs were enriched in module 11, no TFBSs were enriched in module 17, and 1 TFBS was enriched in module 15. No TFBS was enriched in module 36, which was associated with both sex and sex-by-smoking interaction. For the remaining two modules that were associated with sex-by-smoking interaction, 72 TFBSs were enriched in module 02 including estrogen-related receptor alpha (*ESRRA)*, and 7 TFBSs were enriched in module 34. Supplementary Table 8 shows the complete list of enriched TFBSs in each module.

### Comparing the identified sex-by-smoking gene signature with previous literature

Recently, van den Berge *et al*. identified a sex-by-COPD gene expression signature in human whole lung tissue^16^. Since the full gene expression signature is not available, we were only able to compare the top 5 most significant genes for positive sex-by-COPD interaction (*SGMS1, LINGO2, SLC25A1, KDSR, SKP1*) and the top 5 most significant genes for negative sex-by-COPD interaction (*CYorf15A, ZFY, AMPD3, STAP1, MMP13*) against our sex-by-smoking interaction results in airway epithelium.

We first checked these 10 genes in our discovery cohort. At FDR < 0.1, *SGMS1* and *SKP1*, which were down-regulated with COPD in females, were also down-regulated with smoking in females; *KDSR*, which showed both down-regulation with COPD in females and up-regulation with COPD in males, was down-regulated with smoking in females while remaining unchanged with smoking in males. However, none of these genes were replicated in the replication cohort at P < 0.05. Perhaps it is not surprising to see differences between our gene signatures and the gene signature reported by van den Berge *et al*. because the tissues are different (airway epithelium vs. whole lung tissue) and COPD genes are not necessarily the same as smoking genes as we have shown previously^17^.

We also checked our gene signature against the study reported by Engle *et al*.^18^, in which both male and female mice were exposed to smoke or sham (room air). We downloaded the data from the GEO (under accession GSE109776) and performed a differential expression analysis on sex-by-smoke exposure interaction similarly while adjusting for the length of smoke exposure. Of the 184 replicated genes that we identified to be associated with sex-by-smoking interaction in human airway epithelium in our study, only 7 genes were associated with sex-by-smoke exposure in mouse lung tissue in the study of Engle *et al*. at the P < 0.05 threshold.

## Discussion

Studying the transcriptome is a very powerful hypothesis-free approach to elucidate the molecular mechanisms underlying diseases and phenotypes. In this study, we analyzed the differential transcriptomics of human airway epithelium associated with smoking as well as the transcriptomics of smoking differentially between males and females. We found that 1) smoking has a large and reproducible effect on gene expression profiles; 2) the smoking effects on the transcriptome of human airways are modified by the underlying sex of the individual; 3) the sexually dimorphic smoking genes are common throughout the autosome and not restricted to sex chromosomes; and 4) the sexually dimorphic smoking genes are enriched in biological processes that are related to anti-viral responses, type 1 interferon signaling, and autophagy.

There is increasing interest in understanding sexual dimorphisms across organs and tissues. In the study of Melé *et al*.^19^, the authors investigated sexual dimorphism in gene expression data of 1,641 samples from 175 individuals representing 43 sites and identified 753 genes that had tissue specific sex-biased expression (FDR < 0.05). Chen *et al*.^20^ used a much larger sample size (compared to Melé *et al*.) and investigated sexual dimorphism in gene expression data from the GTEx dataset: they evaluated 8,716 human transcriptomes collected from 31 tissues in 549 individuals of whom 188 (34%) were female. The authors observed sexually dimorphic gene expression for 10% of the genes across autosomes in most tissues including the lung. The most sexually dimorphic gene expression was noted in the breast, skin, thyroid, brain, and adipose tissues, while the least dimorphic was in the gastrointestinal tract^20^. Multiple other studies in single cell or tissue types have confirmed to varying extents the large transcription differences between the two sexes^21–24^. Similar patterns emerged from these studies including the presence of large and perhaps underestimated differences in the transcriptome between males and females present throughout the autosomes and not restricted to sex chromosomes. Extrapolating findings from the published studies (even the large ones) to respiratory diseases is challenging since they do not contain data on airway epithelium and lack essential phenotypic information such as smoking. To our knowledge, the current study is the first to report sexually dimorphic gene expression changes in human airway epithelium associated with cigarette smoking. The airway epithelium is the initial site of injury in airway diseases such as COPD and is an important mediator of the host’s immune response to inhaled toxins, allergens and medications^25^.

We found that smoking differentially altered two main pathways in men versus women. These included those related to the host response to viruses (e.g. type 1 interferon signaling) and autophagy. In females, our study revealed that most autophagy genes were up-regulated in response to smoking compared to males where these genes were either down-regulated or only slightly up-regulated (Fig. 6A). The highly-conserved process of autophagy involves the formation of intracellular vacuoles derived from the cell membrane (autophagosomes), which engulf and transport a diverse range of intracellular materials including protein aggregates, organelles, and intracellular bacteria^26^. Although autophagy is considered a normal homeostatic mechanism, its dysregulation has been linked to a number of diseases^27^. In COPD, lung tissue samples show increased vacuolization consistent with upregulated autophagy^28^. However, the role of autophagy in COPD pathogenesis, and its interaction with cigarette smoke (CS) exposure, is complex and may be cell-specific^29^. For example, CS has been shown to induce autophagy-related genes and proteins^28,30^, and increased autophagic flux may contribute to CS-induced epithelial cell death^31^. In contrast, deficiencies in CS-induced autophagy may contribute to CS-induced epithelial cell senescence in COPD^32^ and alveolar macrophages of smokers showed impaired autophagy (which may provide a mechanistic link to increased pulmonary infections in this population)^33^. Nevertheless, we have shown that autophagy-related genes are overall upregulated in women compared to men, in association with the sex-smoking interaction but not sex alone. While sexual dimorphism in autophagy is well-documented^34,35^, our results suggest that sex differences in autophagic processes may be the results of differential responses to CS exposure, rather than sex differences in constitutive autophagy. The functional consequences of our findings for COPD pathogenesis are difficult to predict since (1) the autophagy pathway is complex, and its genetic regulation is poorly understood^36^, (2) it is highly likely that both positive and negative autophagy regulators are differentially expressed^37^, and (3) whether differences in the transcriptome lead to differences in autophagy-related proteins cannot be determined from our study. Our findings should therefore serve as a catalyst for future studies into sex differences in autophagy.

The other sexually dimorphic pathway in response in smoking was type 1 interferon signaling and response to virus. Three of the downregulated genes in females while being unchanged or slightly upregulated in male smokers in Fig. 6B (*IFIT1*, *IFIT2* and *RSAD*) are part of the type 1 interferon signaling pathway and are known to have antiviral activity^38,39^. The *IFIT1* and *IFIT2* genes hinder the expression of viral messenger RNAs by inhibiting the formation of the ribosomal preinitiation complex^40^, while the *RSAD2* gene inhibit multiple DNA and RNA viruses including the human cytomegalovirus^39^ and the human immune deficiency virus (HIV)^41^, hence it is possible that downregulation of *IFIT1*, *IFIT2* and *RSAD* could negatively affect the immune response to viruses in female smokers when compared to male smokers. Previous studies have demonstrated that smoking affects the lung epithelium’s ability to produce the early inductive and amplification phases of the type I interferon response and decreases the ability to elicit an antiviral response^42,43^. Furthermore, these effects were reversible and are almost completely abrogated with the antioxidant glutathione^42^. Sexual dimorphism in the immune response to viruses is well described (reviewed in^44,45^). The reasons underlying this sexually dimorphic response is attributed to sex hormones, sex chromosome-linked genes involved in viral responses, or genes on autosomes that seem to be regulated by sex chromosome-linked genes^44^. Certain sexually dimorphic immune responses are, however, present throughout life, and not necessarily between the years of puberty and reproductive senescence, suggesting that both genes and hormones are involved^45^. In general, females are thought to mount a stronger immune response to viral infections compared to males due to a more robust humoral and cellular immune response resulting in higher male mortality observed in epidemiological studies^46^. This is also consistent with observations that females have a higher risk to many autoimmune diseases^47^.

We performed enrichment analyses in transcription factor binding sites to determine whether or not the observed transcriptional differences are due to response to hormones and as such enriched for hormone receptor binding sites. We observed an enrichment in many transcription factors for the autophagy module including the estrogen-related receptor alpha *ESRRA* (enrichment FDR = 7.02 × 10^−33^) suggesting the biological differences are not only due to the hormonal effect. Further studies are needed to understand the clinical consequences of this sexually dimorphic virus response to smoking with regard to COPD risk and progression, as well as exacerbations attributed to viral and bacterial infections. The finding of down-regulation of anti-viral response pathway genes in females in response to smoking is consistent with the epidemiological observations of higher exacerbation rate in females compared to males^7^.

We have used both gene- and network-level analyses in this study. The network-level approach to transcriptomic data is biologically intuitive since it takes into account the phenomenon of gene co-expression. We have previously used a network approach using WGCNA to identify a reproducible signature for lung function in peripheral blood^48^. The approach allows the identification of “hub” genes that reflect the biological processes of their corresponding modules. For example, the “hub” gene for the virus response enriched module (module 34) is *DDX60* (DExD/H-box helicase 60), a gene that is involved in antiviral response^49^.

The definition of smoking has a big effect on discovery and replication of sexually dimorphic gene expression in response to smoking. When we included the data from GSE37147 (Sterling *et al*.) which included former and current smokers (but no never smokers) for replication, although the sample size is large (n = 238), the replication was poor and hence was not used for replication (Fig. 4D). Similarly, when we included both former and never smokers in our replication cohort vs. current smokers, and despite the increase in sample size, fewer genes were actually replicated compared to when we only included never vs. current smokers (Fig. 4A–C). This is consistent with previous findings from our group highlighting the importance of case definition of smokers on gene signature discovery^50^.

This study has a number of limitations. First, the sample size, although large, may not be sufficient to detect and replicate genes with smaller effects. Second, we used self-reported smoking status based on available data on GEO but self-reports may not accurately reflect smoking habits^50^. Third, we studied changes in airway epithelium given its role in the pathogenesis of lung diseases, but smoking may also affect other cell types such as immune and infiltrating cells in the airways. Finally, changes in gene expression may not necessarily reflect changes in protein levels or activities.

In summary, our study demonstrated that while smoking has a large effect on the transcriptome of airway epithelium, it differentially affects the viral response and autophagy pathways in female smokers compared to males. Such differential expression may explain the increased risk of disease and exacerbation among female smokers. These findings will stimulate mechanistic studies on how sex-specific genes impact the risk of smoking related diseases such as COPD.

## Methods

### Data collection

The search terms “airway epithelium” was used to identify studies of human airway epithelium gene expression from the Gene Expression Omnibus (GEO). After excluding irrelevant studies such as cancer, virus infection, influenza and injury, we identified 18 studies with phenotypic information on subjects’ sex, smoking status and pack-years of smoking. Information on these 18 studies is shown in Supplementary Table 1. Of the studies identified, 16 were reported by the same lab and generated using the same microarray platform (U133A Plus 2.0 array). These datasets were combined together to form the “discovery” set. Phenotypic information such as sex, smoking status, age, COPD status, ethnicity and pack-years were available for 211 subjects (never smokers n = 68; current smokers n = 143) after removing duplicate data and outliers. GSE7895, which was generated using the U133A array, was used as the “replication” set consisting of 21 never smokers, 52 current smokers, and 31 former smokers. Smoking status, age and pack years were available from GEO for GSE7895 but the sex information was missing which was imputed for this cohort based on gene expression data from the discovery cohort (see below). GSE37147, which was generated using the GeneChip Human Gene 1.0 ST Array, was used as an additional replication cohort. Smoking status, sex, pack-years and lung function measurements of forced expiratory volume in 1 second (FEV_1_) and FEV_1_/forced vital capacity (FEV_1_/FVC) were available for 99 current smokers and 139 former smokers. Figure 1 shows a flowchart of the overall study design.

### Data processing

Raw data (.CEL files) were downloaded from GEO for the discovery set, the replication set and GSE37147. Since the 16 studies of the discovery set contained overlapping samples, we carefully examined the phenotypic information of each sample to remove biological and technical replicates. No batch effect was detected among these studies using PCA (Supplementary Fig. 1A). The raw data of the discovery set were then normalized together using the Robust Multi-array Average (RMA) method. Similar to the discovery set, the replication set was also normalized using the RMA method (Supplementary Fig. 1B). The presence of outliers was assessed using a PCA before and after normalization for both the discovery and replication sets. Three outliers were observed and removed from the discovery set (Supplementary Fig. 1C) while no obvious outliers were observed in the replication set (Supplementary Fig. 1D). GSE37147 was also processed similarly and no obvious batch effect or outliers were detected. Large differences in the gene expression profiles were observed among the discovery set, the replication set and GSE37147 (Supplementary Fig. 1E). All data processing and statistical analysis were performed within the R statistical computing environment (version 3.5.0).

### Prediction of sex in the replication set

Since the sex information of the replication set was not available from GEO, we imputed the sex of subjects using gene expression of selected XY-chromosome probes. To identify the probes that best discriminated male from female subjects, elastic net-regularized logistic regression was used for probe selection. The discovery set was partitioned into a training dataset, which contained 70% of female subjects and an equal number of male subjects, and a test dataset, which contained the remaining participants. Models with parameters were built based on the training dataset. The AUC obtained from 100 repeats of a 10-fold cross-validation was used as the criterion to evaluate the performance of the models. The performance of the model with the highest AUC was validated in the testing dataset. Finally, the model was applied to the replication set to predict the sex information.

### Comparing the demographic characteristics

We performed Kruskal-Wallis H test followed by Dunn’s test with Bonferroni correction to compare age and pack-years of smoking between males and females within different smoking status strata in the discovery set, the replication set, and GSE37147.

### Modeling the effect of smoking and sex on gene expression

Linear regression was used to identify probes that were associated with smoking status, sex and sex-by-smoking interaction adjusting for potential covariates such as age, ethnicity and pack-years of smoking in both the discovery and replication sets. The analyses were performed using R package limma^51^. The Benjamini-Hochberg method was used to control the false discovery rate (FDR) and to correct for multiple hypothesis testing. To identify and replicate genes, an FDR < 0.1 was used in the hypothesis-free discovery analyses. For replication, a gene was considered “replicated” if any of the probes showed differential expression between never and current smokers in the replication cohort at a nominal P < 0.05 and with the same direction of effect as in the discovery cohort.

### Weighted gene co-expression network analysis (WGCNA)

The R package WGCNA^48^ was used to construct gene co-expression modules with genes that are highly correlated with each other. The workflow of WGCNA starts with creating a matrix of Pearson correlations between genes, and transforming these into an adjacency matrix through soft thresholding and then converting them into a topological overlap matrix (TOM)^52^. Modules are defined as groups of highly interconnected genes using the average linkage hierarchical clustering to group genes based on the topological overlap of their connectivity, followed by a dynamic cut-tree algorithm to cluster dendrogram branches into gene modules^53^. Each gene within a module will be assigned a Module Membership (MM) whose value ranges between 0 and 1 by relating the gene’s expression profile with the module eigengene determined by the first principal component of the gene expression profiles in that module. Genes with the highest MM are considered “hub” genes, which are thought to be central to the module.

Since the discovery set and the replication set were generated using different microarray platforms, we first summarized the probe-level data of both sets into gene-level data using R package Jetset^54^ before performing WGCNA. Using the 12,537 genes of the discovery set, we identified 37 gene modules and each module was assigned a module number. The assigned module number increases as the size of the module decreases with module 01 holding the largest number of genes. For each of the gene module, an eigengene, which is representative of the module expression profile, was obtained. Linear regression was used to identify module eigengenes that were associated with smoking status, sex, and sex-by-smoking interaction, and COPD status adjusting for ethnicity and pack-years of smoking. To replicate our findings for modules constructed in the discovery set, we calculated their eigengenes in the replication set and then tested for their relationships to smoking status, sex, and sex-by-smoking interaction using linear regression. Similar to the discovery set, the models were adjusted for potential covariates such as pack-years. FDR < 0.1 was used as the threshold for discovery analyses and P < 0.05 was used as the threshold for replication analyses.

### Gene set variation analysis (GSVA)

In addition to the gene- and network-level differential expression analyses, we also performed GSVA^55^ to further replicate our identified gene signatures in the replication cohort. Given a gene set (a list of genes), GSVA calculates a per-subject score for the gene set using a non-parametric unsupervised method and therefore, transforming the data from a gene-by-subject matrix to a gene set-by-subject matrix. Then, differential expression analysis can be performed on the gene set instead of each individual gene to identify the association between the gene set and phenotypes. Linear regression was performed similarly to identify gene sets that were associated with sex-by-smoking interaction. P < 0.05 was used as the threshold for replication of the gene sets. The GSVA analysis was performed using R package GSVA.

### Enrichment analysis

For gene modules that were successfully replicated in the replication set, we performed enrichment analyses using R package clusterProfiler^56^ and TFEA.ChIP^57^ to identify Gene Ontology (GO) biological processes and transcription factor binding sites that were enriched in the module genes.

## Supplementary information

Supplementary Figures

Supplementary Tables
