# Supplementary material for: Widespread Sexual Dimorphism in the Transcriptome of Human Airway Epithelium in Response to Smoking
Source: Sci Rep. 2019 Nov 26;9:17600. doi: 10.1038/s41598-019-54051-y (PMC6879662; doi:10.1038/s41598-019-54051-y)

**Widespread Sexual Dimorphism in the Transcriptome of Human Airway Epithelium in Response to Smoking**

Chen Xi Yang, Henry Shi, Irving Ding, Stephen Milne, Ana I. Hernandez Cordero, Cheng Wei Tony Yang, Edward Kyoo-Hoon Kim, Tillie-Louise Hackett, Janice Leung, Don D. Sin, and Ma’en Obeidat

**Supplementary Data**

**Supplementary Figure 1.** Assessing the presence of batch effect and outliers in the discovery and replication datasets. **(A)** PCA plot on the raw data of the discovery set. **(B)** PCA plot on the raw data of the replication set. **(C)** PCA plot on the normalized data of the discovery set. **(D)** PCA plot on the normalized data of the replication set. **(E)** PCA plot on the combined data of the discovery set, the replication set and GSE37147. Abbreviations used in the figure: NS = Never Smoker, FS = Former Smoke, CS = Current Smoker.


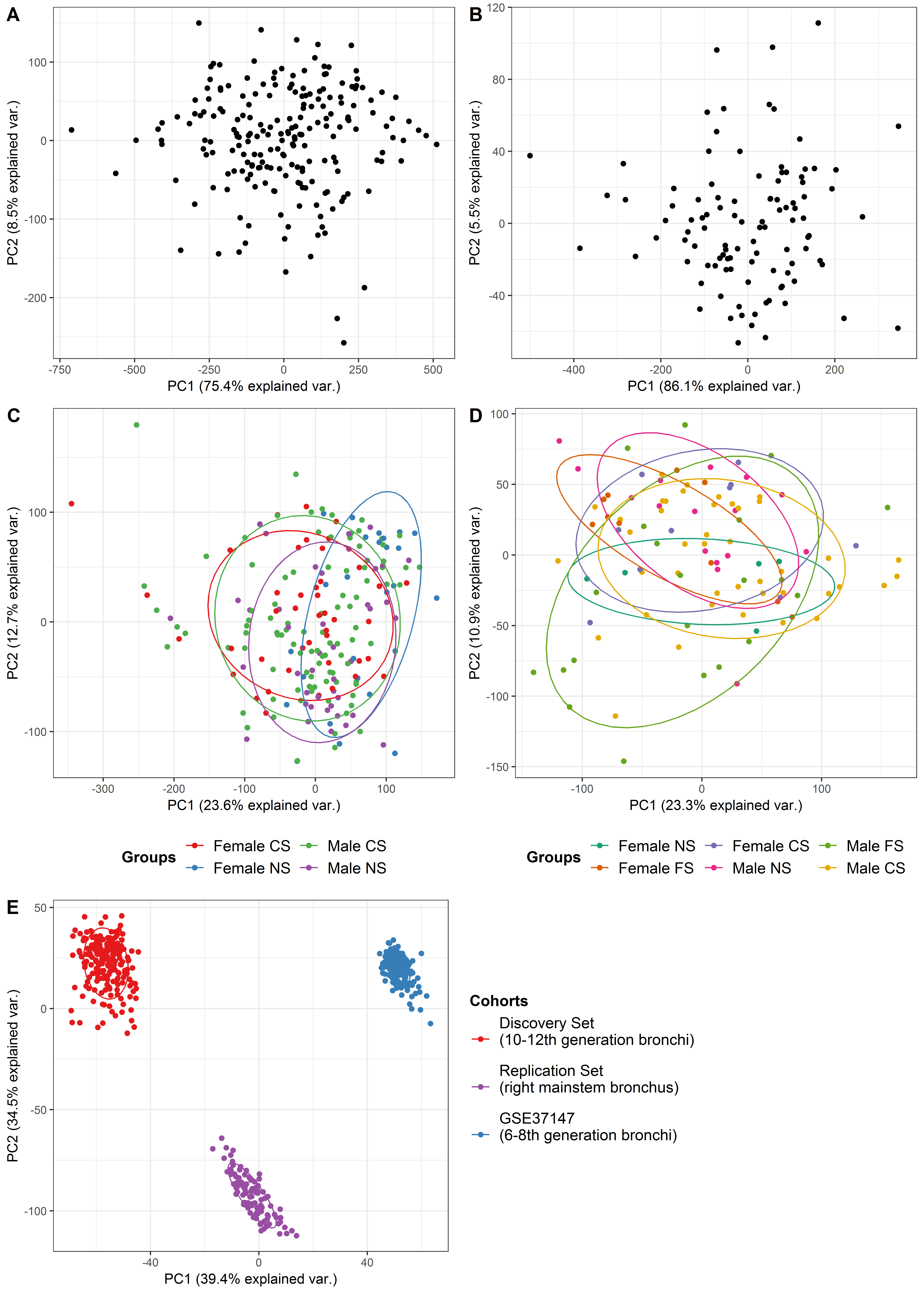


**Supplementary Figure 2**. Prediction of sex in the replication set.

**(A)** Prediction of sex in the testing data of the discovery set. **(B)** Model performance. **(C)** Prediction of sex in the replication set.


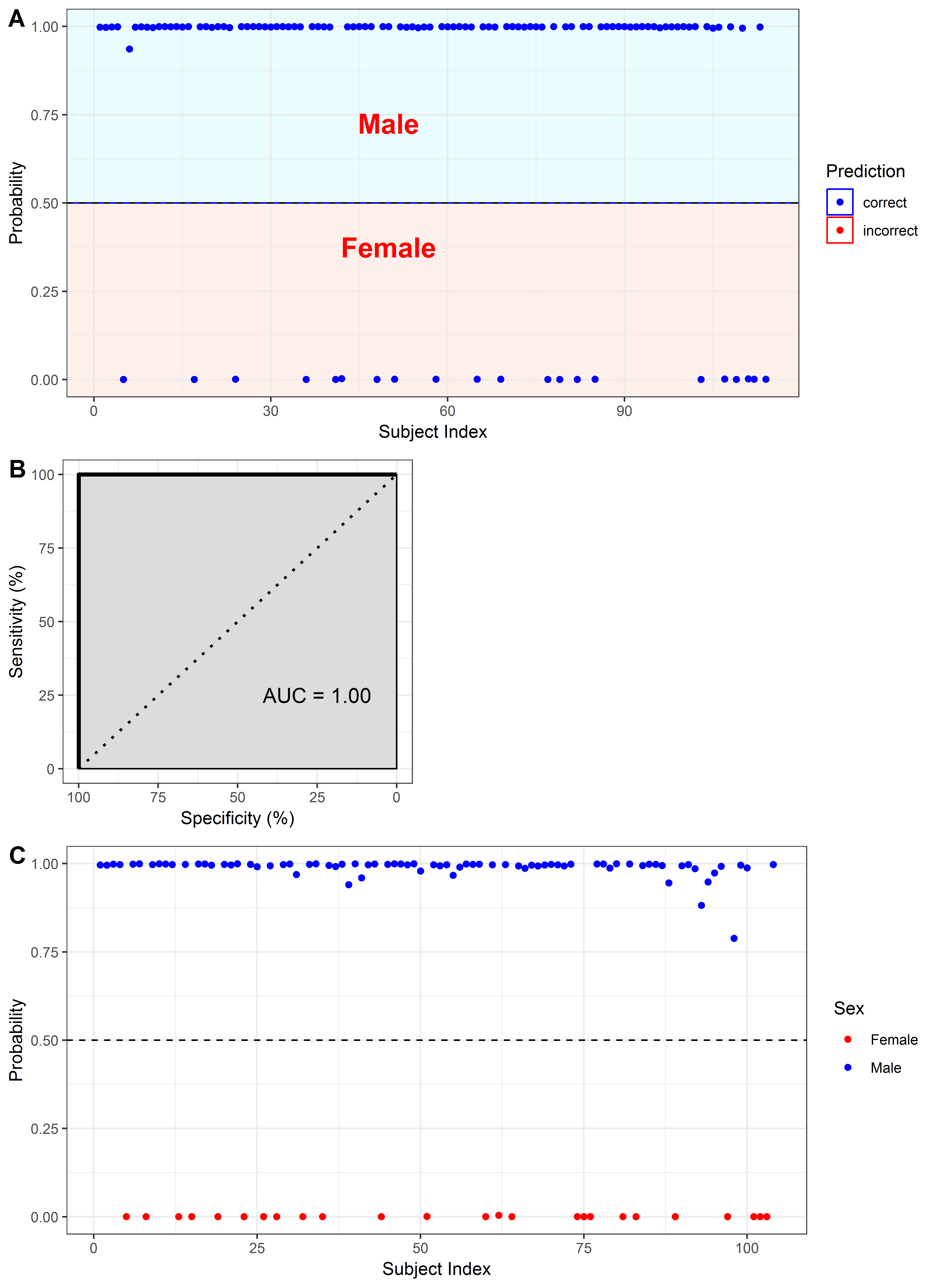

Supplement: Supplementary file 1 — Supplementary Figures [file 41598_2019_54051_MOESM1_ESM.docx]
